# 

**Published:** 1892-12

**Authors:** 


					﻿VOL XII.
DECEMBER, 1892.
NO. 1Z
THE
HOMEOPATHIC PHY^ICIAJI,
A Monthly Journal of Homoeopathic Materia Medica
and Clinical Medicine.
“ He is a freeman whom truth makes free, and all are slaves beside.”
“ Seek the Truth: come whence it may, cost what it wilt.”
EDITED AND PUBLISHED BY
WALTER M. JAMES, M. D.
PHILADELPHIA :
No. 1125 SPRUCE 8TREKT.
LONDON:
ALFRED HEATH & CO., Sole Agents tor Great Britain,
114 Ebury Street, Eaton Square.
TS~\early Subscription, $2.60. invariably in advance. Single numbers, 26 etc.
Entered at the Philadelphia Ped-Offlee at Seoond Claae Mai) Natter.
				

